# Nuclear Progestin Receptor (Pgr) Knockouts in Zebrafish Demonstrate Role for Pgr in Ovulation but Not in Rapid Non-Genomic Steroid Mediated Meiosis Resumption

**DOI:** 10.3389/fendo.2015.00037

**Published:** 2015-03-19

**Authors:** Yong Zhu, Dongteng Liu, Zoe C. Shaner, Shixi Chen, Wanshu Hong, Edmund J. Stellwag

**Affiliations:** ^1^Department of Biology, East Carolina University, Greenville, NC, USA; ^2^College of Ocean and Earth Sciences, Xiamen University, Xiamen, China; ^3^State Key Laboratory of Marine Environmental Science, Xiamen University, Xiamen, China

**Keywords:** knockout, ovulation, meiosis resumption, final oocyte maturation, non-genomic progestin signaling, TALENs, gene editing, progestin receptor

## Abstract

Progestins, progesterone derivatives, are the most critical signaling steroid for initiating final oocyte maturation (FOM) and ovulation, in order to advance fully-grown immature oocytes to become fertilizable eggs in basal vertebrates. It is well-established that progestin induces FOM at least partly through a membrane receptor and a non-genomic steroid signaling process, which precedes progestin triggered ovulation that is mediated through a nuclear progestin receptor (Pgr) and genomic signaling pathway. To determine whether Pgr plays a role in a non-genomic signaling mechanism during FOM, we knocked out Pgr in zebrafish using transcription activator-like effector nucleases (TALENs) and studied the oocyte maturation phenotypes of Pgr knockouts (Pgr-KOs). Three TALENs-induced mutant lines with different frame shift mutations were generated. Homozygous Pgr-KO female fish were all infertile while no fertility effects were evident in homozygous Pgr-KO males. Oocytes developed and underwent FOM normally *in vivo* in homozygous Pgr-KO female compared to the wild-type controls, but these mature oocytes were trapped within the follicular cells and failed to ovulate from the ovaries. These oocytes also underwent normal germinal vesicle breakdown (GVBD) and FOM *in vitro*, but failed to ovulate even after treatment with human chronic gonadotropin (HCG) or progestin (17α,20β-dihydroxyprogesterone or DHP), which typically induce FOM and ovulation in wild-type oocytes. The results indicate that anovulation and infertility in homozygous Pgr-KO female fish was, at least in part, due to a lack of functional Pgr-mediated genomic progestin signaling in the follicular cells adjacent to the oocytes. Our study of Pgr-KO supports previous results that demonstrate a role for Pgr in steroid-dependent genomic signaling pathways leading to ovulation, and the first convincing evidence that Pgr is not essential for initiating non-genomic progestin signaling and triggering of meiosis resumption.

## Introduction

Steroid hormone-regulated physiological processes mediated by steroid hormone receptors that act as transcriptional factors and co-regulators in the nucleus, i.e., genomic steroid signaling pathway, have been well-established. However, many important steroid-dependent physiological processes including meiosis resumption, GnRH release, sexual behavior changes, and sperm activation are too rapid [ranging from a few milliseconds to a few minutes; (1, 2)] to be explained by relatively slow-acting genomic steroid signaling mechanisms, in which responses occur over a time scale of hours to days (3). Physiologically rapid steroid actions typically signal through receptors located on cell membranes or in the cytosol but act independently of transcription and are thus characterized as non-genomic steroid signaling [see Ref. (4) for reference]. Both previously established nuclear steroid receptors and new classes of proteins have been suggested to be the candidate receptor(s) for non-genomic steroid signaling processes (4–9). The role of these receptors as mediators of non-genomic steroid signaling has been vigorously debated but relatively few explicit tests of their function have been conducted. Clearly, further research is required to determine whether these proposed receptors act as non-genomic steroid receptor(s) in specific physiological process.

It is well-known that nuclear progestin receptor (Pgr or nPR) controls various physiological processes including ovulation, breast development, pregnancy establishment, and maintenance via the genomic signaling pathway in mammals (10–14). Our understanding of the role of Pgr in steroid-dependent physiological processes is almost exclusively based on results from the Pgr knockout (Pgr-KO) mouse model (15, 16). In these mutant mice, mature pre-ovulatory follicles fail to release oocytes; there is uterine hyperplasia and inflammation; severely limited mammary gland development; and defects in stereotypical sexual behavior. Evidence concerning the functions of Pgr in non-mammalian models is limited (17) and a non-mammalian Pgr-KO model is still lacking.

In addition to its role in genome-mediated physiological processes, Pgr has also been implicated to mediate rapid and non-genomic progestin signaling in several model systems (18–20). An excellent model for the non-genomic actions of progestin is meiosis resumption during final oocyte maturation (FOM) in fishes and amphibians (4, 21, 22). FOM leads to disappearance of nuclei membrane and clearance of cytoplasm in the mature oocytes. Meiosis resumption is initiated by the binding of progestin to a steroid receptor located on the oocyte surface, which initiates a cascade of non-genomic progestin signaling including rapid down-regulation of cAMP and up-regulation of MAPK. This process does not require the nucleus or transcription, and signaling occurs rapidly at the oocyte surface (4, 21, 22). Overexpression of Pgr in developing oocytes of *Xenopus*, the African clawed frog, accelerated progesterone-induced non-genomic progestin signaling and FOM, implicating Pgr as a candidate non-genomic progestin receptor (23, 24). In contrast, our previous studies have demonstrated that Pgr expression is restricted to the nucleus and cytosol of follicular cells adjacent to the oocytes, but is absent in late developmental stages of the oocytes (stage IV) and sperm in zebrafish (25). This particular pattern of spatial and temporal expression suggests that Pgr may not be involved in the non-genomic progestin signaling and final maturation of germ cells (25, 26).

To better understand the role of Pgr in vertebrate germ cell development, we have generated the first non-mammalian Pgr-KO model in zebrafish using transcription activator-like effector nuclease (TALENS) knockout technology. In comparison to wild-type or heterozygous knockouts, Pgr-KO females show normal oocyte development and maturation but express defects in ovulation and fail to spawn. Our results indicate that Pgr is required for genomic progestin signaling and ovulation, but is not essential for non-genomic progestin signaling and FOM in zebrafish.

## Materials and Methods

### Zebrafish husbandry

Zebrafish used in this study was Tübingen strain that was initially obtained from the Zebrafish International Resource Center and propagated in our lab according to the following procedure. Fish were kept at constant water temperature (28°C), photoperiod (14L:10D, lights on 9:00, lights off at 23:00), pH (7.2), and salinity (conductivity 500–1200 μS) in automatic controlled zebrafish rearing systems (Aquatic Habitats Z-Hab Duo systems, FL, USA). Fish were fed three times daily to satiation with a commercial food (Otohime B2, Reed Mariculture, CA, USA) contained high protein content and supplemented with newly hatched artemia (Brine Shrimp Direct, UT, USA). Fertilized eggs were collected from natural spawning of paired healthy fish in the morning once lights were turned on. Experimental protocols were approved by The Institutional Animal Care and Use Committee at East Carolina University.

### TALEN assembly and *in vitro* synthesis of TALEN mRNAs

**Figure 1:**
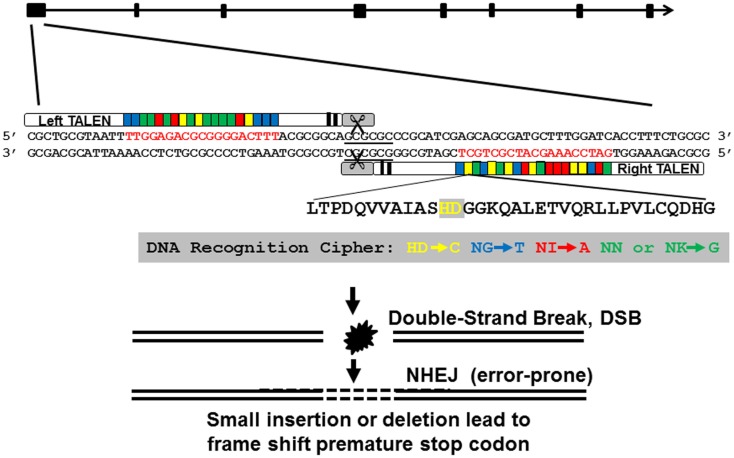
**Genomic structure of nuclear progestin receptor (*pgr*), TALEN targeting sequence, and TALEN-induced mutations**. Exons are shown in filled black boxes, and introns are indicated in black line. TALEN binding nucleotides are in red.

**Figure 2:**
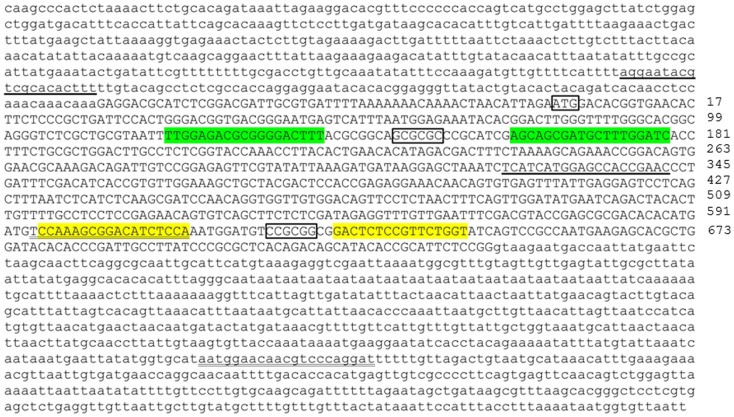
**Genomic DNA sequence of first exon (in upper case) and flanking intron regions (in lower case) of nuclear progestin receptor (nPR or *pgr*)**. First and second TALEN targeting sites are highlighted either in green or yellow, respectively. Translation start site (ATG) and restriction enzyme recognition sites (*Bss*HII: GCGCGC; *Sac*II, CCGCGG) are indicated by box. Forward and reverse PCR primers for amplification of genomic region including TALEN targeting sites are underlined. The numbers on the far right of the figure indicate the positions of the nucleotide counting from the ATG starting site. Transcriptional and translational start sites were manually annotated based on our previous published *pgr* sequence [(25); Genbank access number EF155644] due to annotation errors in databases.

We designed and assembled TALEN molecules using the unit assembly method detailed in Huang et al. (27). First, we retrieved *pgr* mRNA sequence and genomic sequences from NCBI and Ensembl database via ZFIN (www.zfin.org) to determine exon–intron boundaries (Figure 1). Transcriptional and translational start sites were manually annotated based on our previous published *pgr* sequence [(25); Genbank access number EF155644; Figure 2] to correct for annotation errors found in the databases. To identify suitable TALEN target sites, we scanned the coding region of the first exon (785 bp; Figures 1 and 2) and searched for candidate genomic sites using the following parameters: (1) nucleotide T was at position 0; (2) length of the spacer and nucleotides that bound to forward or reverse TALEN protein were between 16 and 22 bp; and (3) a restriction endonuclease site was identified near the center of the spacer for convenient mutation detection and mutation rate estimation. The first target was selected near the beginning of the coding sequence (Figure 2; forward target site sequence: TTGGAGACGCGGGGACTTT, reverse target site sequence: GATCCAAAGCATCGCTGCT, and spacer sequence with a *Bss*HII restriction enzyme site underlined: ACGCGGCAGCGCGCCCGCATCG; Figure 2). The second target site was selected near the end of first exon to generate frameshift mutations leading to a different truncated protein than induced by the first TALEN targeting construct (Figure 2; forward target site sequence: CCAAAGCGGACATCTCCA, reverse target sequence: ACCAGAACGGAGAGTC, and spacer sequence with a *Sac*II restriction enzyme site underlined: AATGGATGTCCGCGGCG). All assembled TALEN vectors were confirmed using Sanger sequencing.

These assembled TALEN vectors were linearized with *Not*I, gel extracted, and purified using QIAquick gel extraction kit according to manufacturer’s specifications (Qiagen, MD, USA), and mRNAs transcribed using SP6 mMACHINE kit (Ambion, TX, USA). The transcribed mRNAs were stored at -80°C until use. Immediately prior to microinjection, mRNA was diluted into workable concentrations (100 ng/μl) with nuclease-free water, and mixed with an equal volume of 0.5% phenol red solution (Sigma P0290).

### Microinjection, mutation analyses, and mutant lines establishment

To generate a founder population (F0), fertilized eggs were collected within 5 min of natural spawning of wild-type fish from their crossing tanks that were set up the night before. Approximately 1 nl of TALEN transcripts (100 ng/ul) was microinjected into fertilized eggs between the 1–4 cell stages using a glass microcapillary pipette attached to a micromanipulator under a stereomicroscope (Leica MZ6). Injection was driven by compressed N_2_ gas, under the control of a PV820 Pneumatic PicPump (World Precision Instrument, FL, USA). For comparison and to estimate mutagenesis efficiency, uninjected wild-type zygotes were also collected and incubated in parallel to the injected embryos. A pool of genomic DNA was extracted from 30 normally developing wild-type or TALENs-microinjected embryos at 2dpf (day post fertilization) using a HotSHOT method (28). In short, embryos were incubated in 200 μl hot alkaline solution (50 mM NaOH) for 20 min at 95°C. Then, we amplified DNA regions containing the *pgr* TALEN target site 1 or 2 using PCR. The PCR reaction mixture (20 μl) includes 4 μl of 5 × PCR buffer, 2 μl 25 mM MgCl_2_, 0.4 μl of 10 mM dNTP, 0.3 μl of genomic DNA extract, 0.1 μl (0.5 U) of *Taq* DNA polymerase (Promega, Catolog #M8295), and 0.2 μl of forward or reverse primer (10 pmol/μl, site1 forward: 5′- AGGAATACGTCGCACACTTT-3′, site 1 reverse: 5′- TGGAGATGTCCGCTTTGGA -3′; site 2 forward: TCATCATGGAGCCACCGAAC, site 2 reverse: ATCCTGGGACGTTGTTCCATT). The PCR conditions were as follows: 2-min denaturation at 94°C, 36 cycles of 30 s denaturation at 95°C, 30 s annealing at 58°C, and 1 min elongation at 72°C; final 10min elongation at 72°C. Amplicon size and sequence were then confirmed by gel electrophoresis and Sanger sequencing. About 8.2 μl of PCR product was digested with 0.8 μl of *Bss*HII (5 U/μl) or *Sac*II (20 U/μl) (NEB, Cambridge, MA) in 10 μl volume with 1 μl of 10 × reaction buffer at 37°C for 2 h. Mutation rates were estimated by comparing band intensities of undigested PCR products (due to loss RE sites caused by mutation) to intensities of digested PCR products (due to retention of RE sites, i.e., wild-type). The percentage of uncleaved band (i.e., potential mutations in target site) was measured by Image J software. The uncleaved bands were recovered after gel electrophoresis and cloned into a TA cloning vector (29) for sequencing analysis. For each TALEN treatment, about 10 recombinant PCR-amplicon-containing colonies were sequenced to determine mutation types (in frame vs. frameshift).

**Figure 3:**
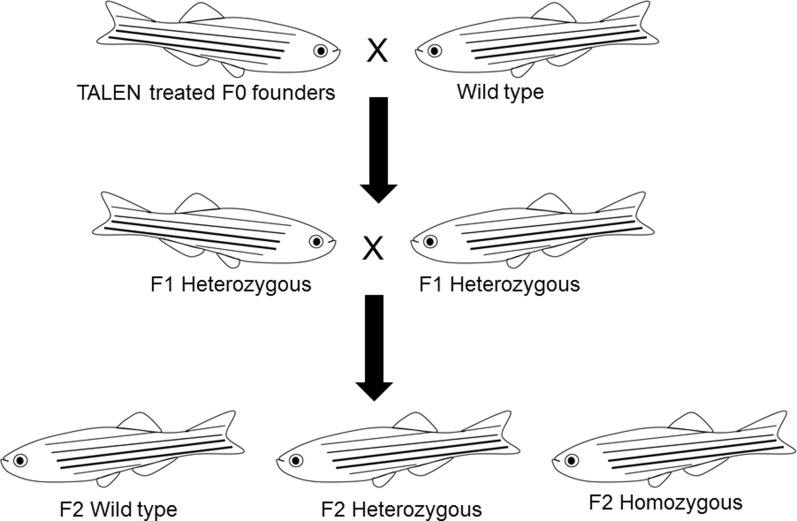
**Schematic drawing shows the procedure used to generate Pgr homozygous mutant lines**.

**Figure 4:**
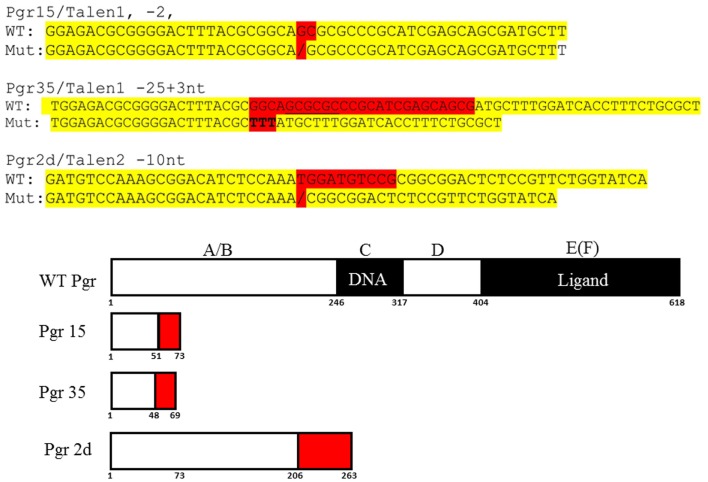
**DNA and predicated amino acid sequences of three Pgr-null allele fish lines**. Nucleotides mutated are highlighted in red and yellow indicates flanking sequences. Numbers under the box indicate amino acid number from the translation start site.

To identify germline-transmitted mutations, the F0 founder embryos were raised to adulthood and outcrossed with wild-type fish. Genomic DNA from each cross was extracted from 30 randomly selected and pooled F1 embryos, and the status of the target site was analyzed *via* PCR amplification, restriction enzyme digestion, and DNA sequencing as described above. The remaining F1 embryos with identified frame shift mutations were raised to adulthood and were genotyped individually. Genomic DNA was extracted from part of the caudal fin of adult fish in a 50 μl hot alkaline solution and analyzed as above. Heterozygous F1 adults carrying the same frameshift mutant alleles were crossed to each other. These crosses yielded wild-type, heterozygous, and homozygous F2 fish that were further characterized genetically and physiologically (Figure 3). Two mutant lines with different frame shift mutations were generated using TALENs directed toward target site 1 and one frame shift mutant line was generated using TALENs directed toward target site 2 (Figure 4).

### Spawning and fertility

After F2 fish reached maturity, wild-type, heterozygous, and homozygous mutants were genotyped and separated based on restriction enzyme and sequence analyses of genomic DNA extracted from tailfin clipping as described in the previous section. Fish homozygous for mutant alleles were crossed and fertility was determined based on spawning occurrence and viable offspring. When no spawning occurred in a minimum of six trails with specific mating pairs, reciprocal crosses between males and females bearing homozygous and heterozygous mutations for null alleles or wild-type were conducted to determine whether the observed reproductive defects were influenced by gender.

### Ovulation, final oocyte maturation, and histological analyses

Ovary morphology and histology were assessed to determine the cause of infertility in females homozygous for TALEN 1 or 2-null alleles. At least 10 homozygous Pgr-KO, heterozygous null allele bearing or wild-type siblings were sampled at 9:00 immediately upon exposure to a lighted room. Fish were euthanized by severing the spinal cord followed by an overdose of MS-222 (200 mg/l in buffered solution). Body weight, body length, and gonad weight were measured and recorded. Development of oocytes, i.e., occurrence of final maturation or ovulation, were visually identified and digitally photographed. For histological analyses, ovaries were collected at either 10:00 or 22:00, 1 h after lights on or 1 h prior to lights off, and fixed in 10% buffered formalin for 12 h, dehydrated through increasing concentrations of ethanol, and embedded in paraffin. A series of 8-μm sections were made and were subjected to hematoxylin and eosin (HE) staining or immunohistological staining using Pgr specific antibodies developed previously in our lab (25).

### *In vitro* incubation

To determine whether *in vivo* anovulation in Pgr-KO female fish was due to a lack of Pgr-mediated signaling locally in follicular cells, ovaries were collected from Pgr-null or wild-type fish in the evening time around 22:00 when few oocytes mature spontaneously, and *in vitro* incubation of ovaries was conducted (26). Ovaries were removed from fish immediately following an appropriate anesthetic overdose (MS-222: 200 mg/l in buffered solution) and placed in an incubation medium (50% L-15 media, Sigma) containing 15 mM HEPES (pH 7.2). Ovaries were cut into small pieces weighing approximately 20 mg each, which were incubated separately in 1 ml of incubation medium in a 24-well plastic culture dish. Steroid (4-pregnen-17, 20β-diol-3-one: i.e., DHP, final concentration 100 nM) was dissolved in ethanol, and human chronic gonadotropin (5 U/ml) was dissolved in incubation medium. Ten microliters of hormone solution, incubation medium, or ethanol as control was added to individual ovary slices in separate wells of the ovary culture. The ovarian culture incubation was conducted overnight at room temperature (24°C). Occurrence of FOM and transition to stage V mature oocytes were determined by the increase in optical clarity of the cytoplasm and disappearance of the nuclear membrane (the germinal vesicle breakdown: GVBD) as viewed under a dissecting microscope. Ovulation was defined by the separation of individual stage V mature oocytes from immature clustered oocytes and absence of surrounding follicular cells in these oocytes; whereas anovulation was defined by the presence of stage V mature oocytes within clustered immature oocytes and accompanied follicular cells in these mature oocytes, and absence of individual dispersed stage V mature oocytes.

### Confocal microscope imaging

To determine whether mature stage V oocytes were still enclosed within the follicular cells in wild-type and mutant fish, fluorescent confocal microscopic analysis of follicular nucleus staining was conducted. Stage V mature oocytes that completed final oocyte maturation from wild-type or Pgr-KO fish were isolated, fixed in 10% buffered formalin for at least 30 min, incubated for 5 min in DAPI nuclear stain (10 μg/ml) in Cortland’s medium, and then imaged using an inverted-fluorescent-spin-disk-confocal-scanning microscope (model: IX2-DSU, Olympus America, Center Valley, PA, USA).

## Results

### Generation of Pgr-KO zebrafish model

**Figure 5:**
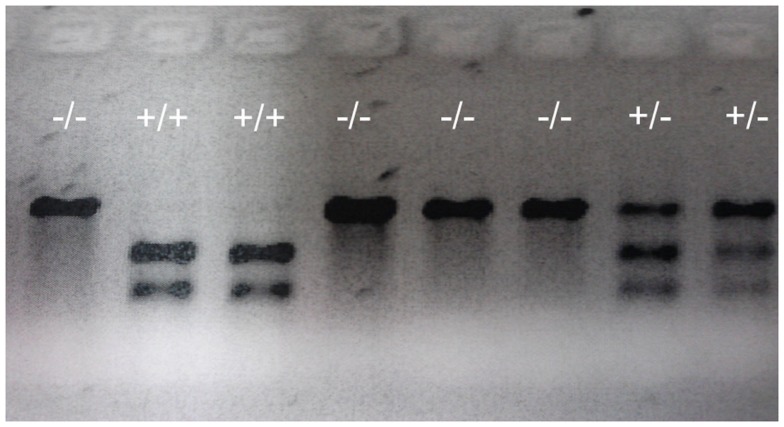
**Representative genotypes of F2 adult fish**. A DNA fragment (769 bp) was PCR amplified from genomic DNA extracted from part of the tailfin clipped from F2 adult fish, then analyzed using restriction enzyme digestion. PCR fragments from wild-type (indicated by +/+ sign) that retains the restriction enzyme site were digested into two fragments (305 and 466 bp). PCR fragments from homozygous mutants (indicated by -/- sign) in which the restriction enzyme site was destroyed were undigestible and therefore retained the original full length 769 bp fragment. Digests of PCR fragments from heterozygotes (indicated by +/- sign) have both digestible and undigestible bands.

**Figure 6:**
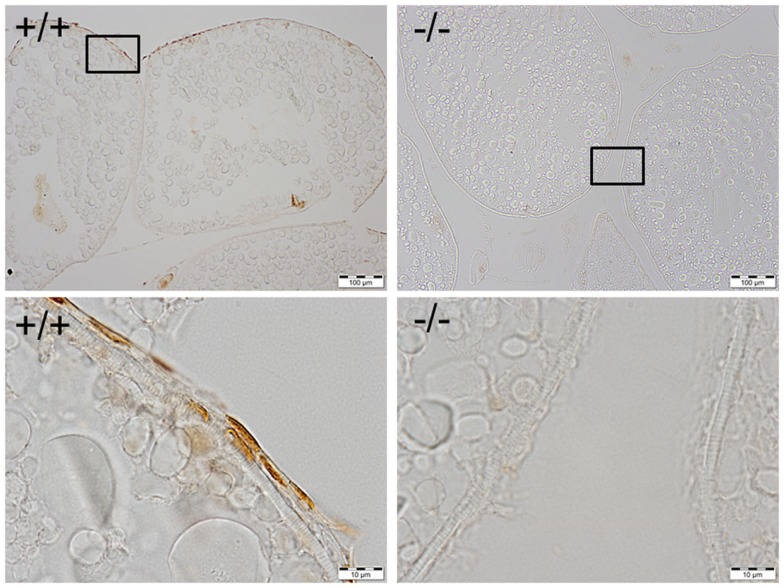
**Immunohistochemical staining of Pgr was observed in the nucleus of follicular cells of wild-type fish (left side), while no staining was observed in the follicular cells of Pgr-KO fish (right side)**. The ovaries were collected and fixed at 22:00, 1 h prior to lights off, when Pgr was expressed in the wild-type fish. Pictures located in the bottom panel are magnified images of the areas outlined in the top panel.

Based on the analyses of restriction enzyme digestion and DNA sequence of genomic DNA extracted from a pool of 30 embryos at 2dpf microinjected with TALEN mRNAs, both pairs of TALENs effectively (mutagenesis efficiency: 30–60%) induce insertion/deletion mutations (data not shown). Comparison of the wild-type and TALENs-induced mutant sequences demonstrated two nucleotides (GC at nucleotide positions 146 and 147 from ATG starting site, Figures 2 and 4) were deleted in Pgr 15 mutant line, which caused a +1 translational frame shift predicted to generate a truncated peptide lacking amino acids 74–617 (from Methionine starting site; Figures 2 and 4) with concurrent alteration of amino acids 51–73(Figures 2 and 4). Additional comparisons of the wild-type and TALENs-induced mutations from Pgr line 35 showed a 25 nucleotide deletion (GGCAGCGCGCCCGCATCGAGCAGCG, positions 142–166, Figures 2 and 4) coupled with a three nucleotide (TTT) insertion. This mutation led a +2 frame shift, and resulted in a truncated peptide lacking amino acids 70–617 with concurrent alteration of amino acids 48–69. TALENs directed toward target site 2 generated a 10 nucleotide deletion (TGGATGTCCG, nucleotide positions 616–625, Figures 2 and 4) that resulted in truncation of amino acids 263–617 and alteration of amino acids 206–263 in the coding sequence (Figure 4). These F1 embryos were raised to maturity, genotyped, and intercrossed to produce F2 homozygotes (Figure 3), heterozygotes, and wild-type siblings, which were also raised to maturity, genotyped (Figure 5), and used for further experiments. Specific immunostaining of Pgr was observed in the follicular cells of wild-type fish, but not in the follicular cells of Pgr-KO fish (Figure 6), suggesting Pgr was successfully knocked out. No significant difference in body length, body weight, and gonad weight were observed in comparisons between Pgr-KO and wild-type fish (data not shown).

### Pgr-KO females fail to spawn

**Figure 7:**
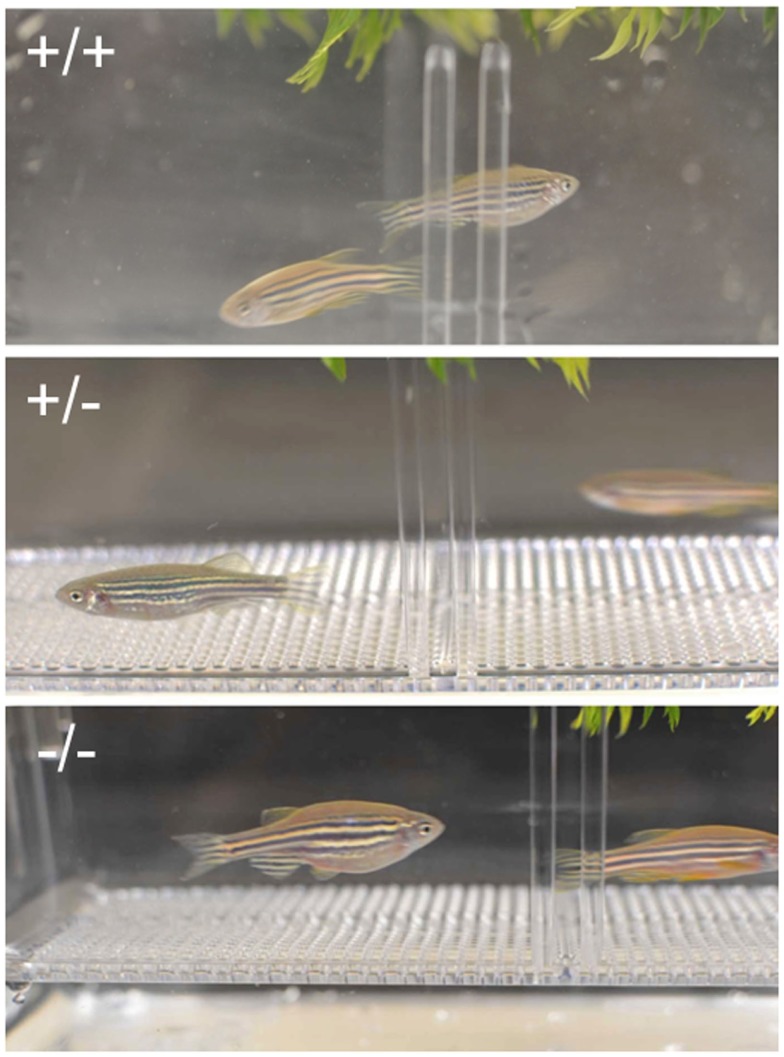
**Crossing of wild-type (+/+, top panel), heterozygous (+/-, middle panel), and homozygous (+/+, bottom panel) females and corresponding males**. No obvious external morphological difference was observed between fish with different genotype, but homozygous Pgr-KO fail to spawn.

No spawning was observed in multiple trials (minimal six trails for each pair) and multiple pairs (minimal six pairs per mutant line) when homozygous Pgr-null females were paired with either Pgr null or wild-type males. In contrast, spawning was observed when heterozygous females were paired with wild-type, heterozygous, or even homozygous mutant males (Figure 7). Gentle abdominal squeezing of homozygous Pgr-KO females also failed to extrude any mature oocytes, which typically could be obtained through abdominal squeezing of wild-type or heterozygous females.

### Pgr-KO female fish undergo FOM but do not ovulate

**Figure 8:**
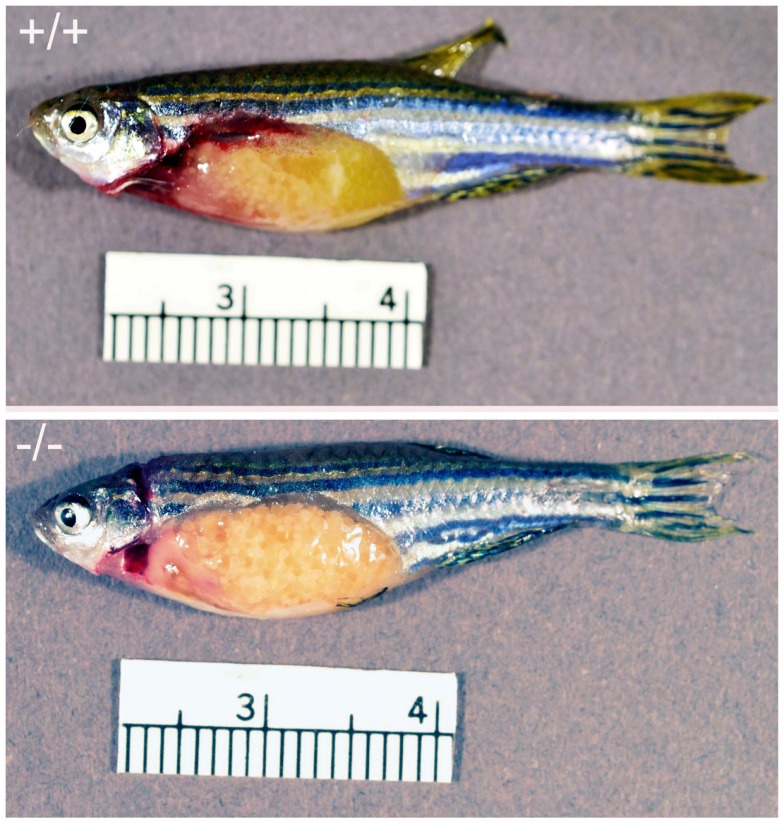
**Comparison of ovary from Pgr-KO fish (-/-, bottom picture) to that of wild-type fish (+/+, top picture) sampled in the early morning after the onset of illumination**. The majority of the gut contents are yellowish colored oocytes. Ovulated and transparent mature oocytes were located on the top right side of the wild-type fish ovary ready to be spawned, which were separated from immature oocytes that were located at the bottom left side of the ovary. In comparison no ovulated oocytes were observed in the Pgr-KO females. Instead, transparent and mature oocytes were scatted around and trapped within the ovary in Pgr-KO females.

**Figure 9:**
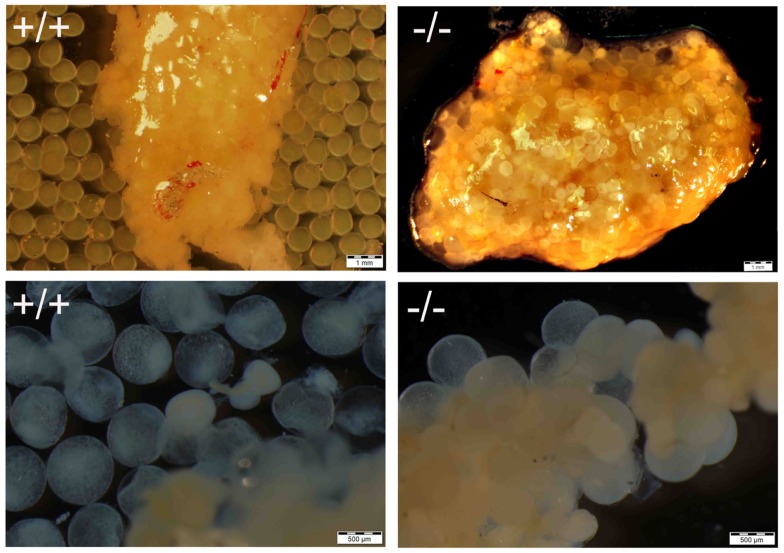
**Comparison of maturation and ovulation between wild-type (+/+) and Pgr-KO (-/-) *in vivo* and *in vitro***. Ovaries were collected at 9:00 following the onset of illumination (top panel, matured *in vivo*), or ovarian fragments were cultured overnight *in vitro* (bottom panel, matured *in vitro*). Individual mature oocytes were released from the ovary into the medium when removed from wild-type fish (top left) or cultured overnight in ovarian fragments (bottom left), while mature and transparent oocytes were trapped within the entire ovary (top right) or attached the ovarian fragment (bottom right) in Pgr-KO ovary.

**Figure 10:**
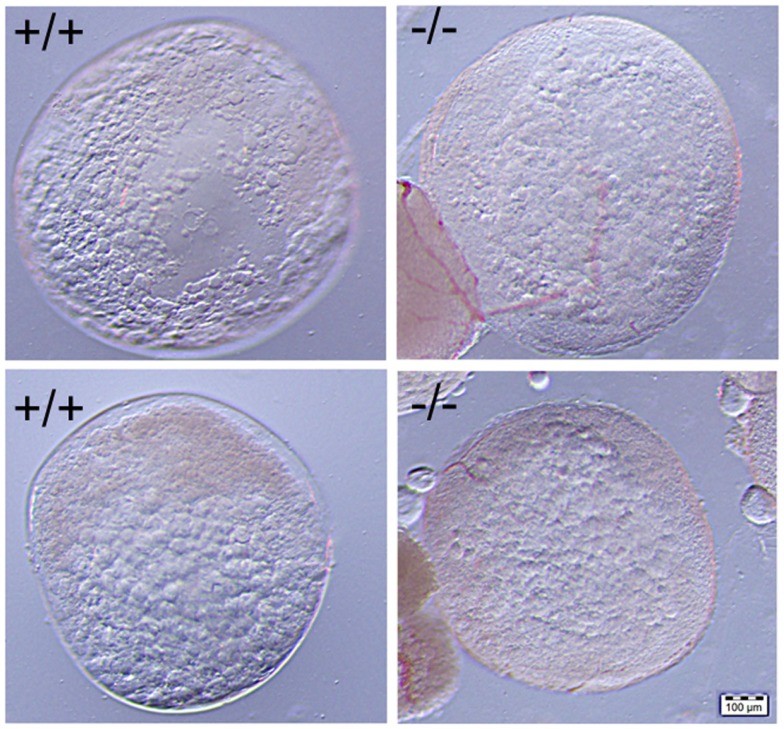
**No significant difference was observed in the comparison of mature stage V oocytes of wild-type (+/+, left side panel) to those of Pgr-KO (-/-, right side panel)**. Cytoplasm of the oocytes becomes transparent when fully-grown immature oocytes underwent germinal vesicle breakdown (GVBD) and final oocyte maturation (FOM) in wild-type fish (left side) or Pgr-KO fish (right side) obtained *in vivo* (top) or *in vitro* (bottom). Partial images of immature and opaque oocytes attached to stage V oocytes can be observed at the left corner on the right side panel.

**Figure 11:**
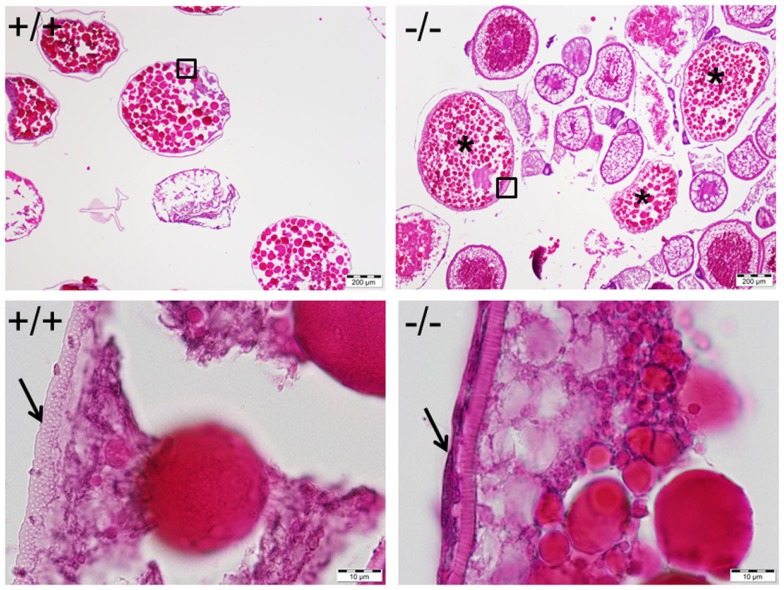
Mature and anovulated stage V oocytes (indicated by *) containing follicular cells (indicated by an arrow in bottom right) were observed and trapped within the ovary of the Pgr-KO fish (-/-, right side panel). In comparison, mature and ovulated stage V oocyte from wild-type fish (+/+, left side panel) contains no follicular cells. Ovaries were collected and fixed at 10:00, 1 h after the onset of illumination, when mature oocytes were ovulated in wild-type fish. Pictures located at bottom panel are magnified images of the areas outlined in the top panel.

**Figure 12:**
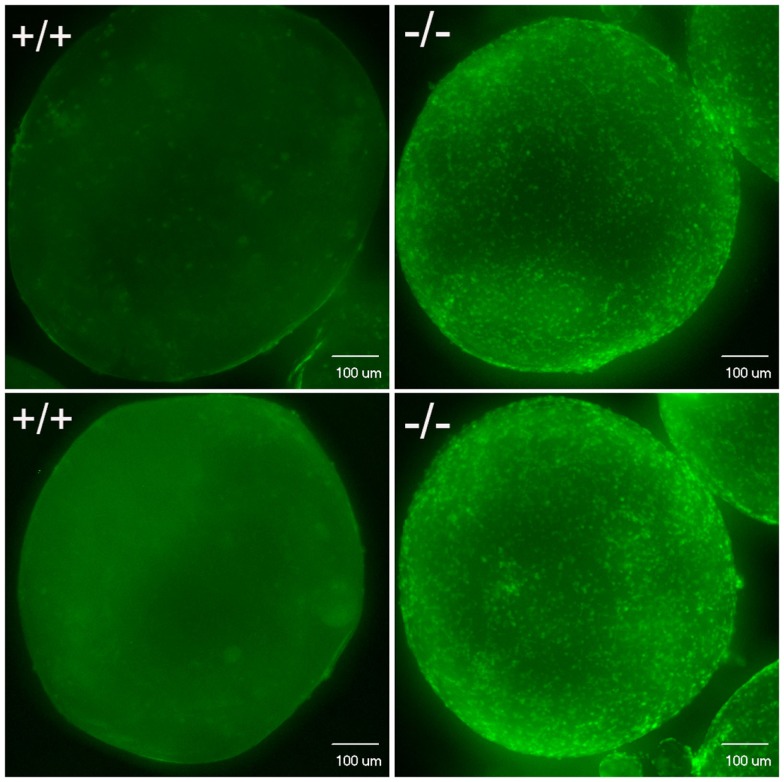
**Confocal image analysis of individual mature oocytes to determine the occurrence of ovulation based on whether stage V mature oocytes contain DAPI nuclear stained follicular cells**. Mature stage V oocytes obtained *in vivo* (top) or *in vitro* (bottom) from wild-type fish (+/+, left side) showed only background staining, which indicates these oocytes lack follicular cells. In contrast, mature stage V oocytes from Pgr-KO fish (-/-) isolated from ovaries *in vivo* (top) or *in vitro* (bottom) showed distinct nuclear staining of the follicular cells, which indicates these mature oocytes still contain follicular cells.

**Figure 13:**
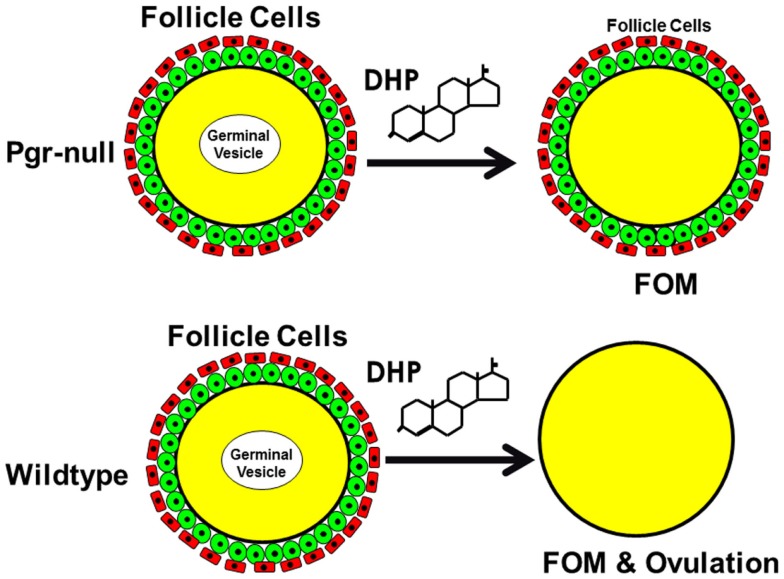
**Schematic drawing shows nuclear progestin receptor (Pgr or nPR) is required for ovulation but not for oocyte maturation**. Progestin is able to induce final oocyte maturation (FOM) but not ovulation in Pgr-KO female fish, while progestin induces both FOM and ovulation in wild-type fish.

Examination of surgically removed ovaries from Pgr-KO homozygotes early in the morning revealed that oocytes developed and matured normally from fully grown stage IV immature oocytes to stage V mature oocytes (Figures 8–12). Two typical characteristics, i.e., clearance of cytoplasm and disappearance of the nuclear membrane were observed in Pgr-KO oocytes as well as in wild-type fish (Figures 8–12). No significant difference was observed under a dissection microscope comparing stage V mature oocytes that underwent FOM from Pgr-KO fish to those from wild-type fish (Figure 10). However, histology and confocal image analyses indicated fully mature stage V oocytes were trapped within the follicular cells and within the ovary, and did not ovulate (Figures 11 and 12). In contrast, fully developed stage V mature oocytes were ovulated and released from follicular cells and the ovaries of wild-type (Figures 8–13) or heterozygous mutant fish (same as wild-type, data not shown). The observation of stage V mature oocytes trapped within the ovaries provides strong evidence that Pgr null allele homozygotes can undergo FOM *in vivo*. *In vitro* studies using treatments that promote maturation and ovulation, i.e., human gonadotrophin (HCG) or the maturation inducing steroid DHP, also indicate that the fully grown immature oocytes also underwent FOM, but fail to ovulate. By comparison, oocytes from heterozygous or wild-type females underwent both FOM and ovulation after *in vitro* treatment of ovarian tissue with HCG or DHP (Figures 9,10,12,13).

## Discussion

It is well-established that a progestin-dependent non-genomic steroid signaling mechanism that acts through an oocyte surface receptor is required for meiosis resumption and the final oocyte maturation in fish and amphibians (4, 21, 22). The identity of all the receptor(s) that mediate non-genomic progestin action in the oocyte remains unclear (4, 5, 23, 24, 30). Previous studies had used overexpression or knockdown of these candidate receptors, which have sometimes lead to contradictory results (23, 24, 30). Loss-of-function and knockout studies are currently lacking in the zebrafish and *Xenopus* models. Using TALEN technology, we have generated the first non-mammalian model for Pgr-KO and provided the first evidence that Pgr is required for the activation of ovulation via a steroid-dependent genomic signaling mechanism in fish, but not for the initiation of non-genomic progestin signaling (4, 21, 22) and FOM (Figure 13).

It is now generally accepted that steroids are involved directly in physiological processes that occur very rapidly via a non-genomic signaling pathway(s) that are initiated at or near the cell surface. This steroid-dependent rapid and non-nuclear-mediated non-genomic signaling mechanism has been shown in various systems [see Ref. (4) for detailed reference]. Despite general agreement that non-genomic steroid-dependent systems play a direct role in rapid physiological responses, the identity of the membrane receptor mediating a particular rapid steroid response often remains unclear and thus hampers advancement in the field of non-genomic steroid signaling and actions. Several different classes of proteins including nuclear steroid receptor have been hypothesized as non-genomic steroid receptor candidates (4, 5). To date, these studies have relied heavily on the measurement of intracellular signaling or the expression of the candidate proteins either in cells lines or mammalian cancer lines. In order to elucidate the identity of these non-genomic steroid receptors, it is critical to establish knockout animal models with null alleles for candidate genes and to study their effects on physiological processes controlled by non-genomic steroid signaling. In zebrafish homozygous Pgr-KO, we observed FOM and anovulation, and therefore concluded that while Pgr plays a role in a genomic signaling pathway and ovulation, but it is not required for non-genomic signaling and FOM.

Our previous studies demonstrated that the transcript and protein product of Pgr were restricted to the follicular cell layer of fully grown immature oocytes and increased prior to oocyte maturation and ovulation, but were absent in the oocytes in zebrafish (25, 26). Pgr expression is similarly restricted exclusively to granulosa cells of preovulatory follicles in all mammalian species studied to date. Pgr is primarily localized to the nuclei and cytosol of murine granulosa cells and is undetectable in cumulus cells or oocytes in mammals (12, 13, 16). Despite the absence of Pgr in the target cell, i.e., Pgr in oocytes, progestin-induced oocyte maturation still occurs in fishes and amphibians, which suggests that Pgr plays no direct role in mediating this rapid non-genomic physiological process (5, 31). Results reported in this study that show fully grown immature oocytes from Pgr-KO fish undergo FOM both *in vivo* and *in vitro* provide clear evidence that Pgr is not required for the non-genomic progestin signaling leading to meiosis resumption in zebrafish. Receptor(s) other than Pgr, such as mPRs, are likely required for the non-genomic progestin signaling and meiosis resumption in zebrafish (26, 30, 32).

The critical role of Pgr in ovulation has been demonstrated in mammals using Pgr antagonists and a mouse gene knockout model (11, 13–15). Normal growth, development and maturation of follicles, and oocytes were observed in mouse Pgr knockouts and it was observed that mature oocytes are entrapped within follicles, leading to an anovulatory phenotype (12, 15). We observed the same phenotype in Pgr knockout zebrafish, which demonstrates a conserved function of Pgr in ovulation among vertebrates. Pgr is an important regulator of gene transcription, specifically of genes found to be necessary for successful oocyte release from the preovulatory follicle (11, 12). Pgr-regulated genes identified to date include those coding for proteases, growth factors, signal transduction components and transcription factors, but few have been demonstrated to play a direct role in ovulation. These Pgr-regulated genes in granulosa cells also lack Pgr binding response elements in their upstream promoters, and therefore unique transcriptional mechanisms are believed to play a role [see Ref. (12) for review]. Our Pgr-null zebrafish model thus provides a unique opportunity for identifying conserved genes and networks regulated directly by Pgr in vertebrates.

## Author Contributions

YZ conceived and supervised the project, performed experiments, analyzed results and wrote the paper. DL performed TALEN synthesizing and mutation screening. ZCS performed TALEN synthesizing, mutation screen and spawning experiment. SC and WH supervised the project and discussed the results. EJS discussed the results and wrote the paper.

## Conflict of Interest Statement

The authors declare that the research was conducted in the absence of any commercial or financial relationships that could be construed as a potential conflict of interest.
